# Genetic Differentiation and Genetic Diversity of *Castanopsis* (Fagaceae), the Dominant Tree Species in Japanese Broadleaved Evergreen Forests, Revealed by Analysis of EST-Associated Microsatellites

**DOI:** 10.1371/journal.pone.0087429

**Published:** 2014-01-30

**Authors:** Kyoko Aoki, Saneyoshi Ueno, Takashi Kamijo, Hiroaki Setoguchi, Noriaki Murakami, Makoto Kato, Yoshihiko Tsumura

**Affiliations:** 1 Graduate School of Human and Environmental Studies, Kyoto University, Kyoto, Kyoto, Japan; 2 Department of Forest Genetics, Forestry and Forest Products Research Institute, Tsukuba, Ibaraki, Japan; 3 Faculty of Life and Environmental Sciences, University of Tsukuba, Tsukuba, Ibaraki, Japan; 4 Makino Herbarium, Tokyo Metropolitan University, Hachioji, Tokyo, Japan; CNR, Italy

## Abstract

The broadleaved evergreen forests of the East Asian warm temperate zone are characterised by their high biodiversity and endemism, and there is therefore a need to extend our understanding of its genetic diversity and phylogeographic patterns. *Castanopsis* (Fagaceae) is one of the dominant tree species in the broadleaved evergreen forests of Japan. In this study we investigate the genetic diversity, genetic structure and leaf epidermal morphology of 63 natural populations of *C. sieboldii* and *C. cuspidata*, using 32 Expressed Sequence Tag associated microsatellites. The overall genetic differentiation between populations was low (*G*_ST_ = 0.069 in *C. sieboldii* and *G*_ST_ = 0.057 in *C. cuspidata*). Neighbor-joining tree and Bayesian clustering analyses revealed that the populations of *C. sieboldii* and *C. cuspidata* were genetically clearly differentiated, a result which is consistent with the morphology of their epidermal cell layers. This suggests that *C. sieboldii* and *C. cuspidata* should be treated as independent species, although intermediate morphologies are often observed, especially at sites where the two species coexist. The higher level of genetic diversity observed in the Kyushu region (for both species) and the Ryukyu Islands (for *C. sieboldii*) is consistent with the available fossil pollen data for *Castanopsis*-type broadleaved evergreen trees during the Last Glacial Maximum and suggests the existence of refugia for *Castanopsis* forests in southern Japan. Within the *C. sieboldii* populations, Bayesian clustering analyses detected three clusters, in the western and eastern parts of the main islands and in the Ryukyu Islands. The west-east genetic differentiation observed for this species in the main islands, a pattern which is also found in several plant and animal species inhabiting *Castanopsis* forests in Japan, suggests that they have been isolated from each other in the western and eastern populations for an extended period of time, and may imply the existence of eastern refugia.

## Introduction

The Quaternary climate cycles played an important role in shaping the distribution of biodiversity among current populations, even in warm-temperate zones, where the land was not covered by ice sheets [1], [2]. Phylogeographic patterns established by analyzing genetic variation in contemporary organisms have proven highly informative in determining the glacial and postglacial demographic histories of individual species [1], [3]. Avise [3] proposed that comparing genetic data from multiple co-distributed taxa could be useful in elucidating the relative influences of major historical events on current patterns of biodiversity. Such studies have been conducted for several areas of the world, including Europe [1], [4], North America [5], [6], and Asia (with a particular focus on Japan) [7]–[13]. In the current study, we focused on the *Castanopsis* (Fagaceae)-type broadleaved evergreen forest community in Japan, which characterizes the biodiversity and endemism of the East Asia. We aimed to elucidate the effects of past climatic changes more clearly on the current genetic diversity of the species that inhabit warm-temperate zone, because the effects of climate change are particularly severe for these members of the community.

**Figure 1:**
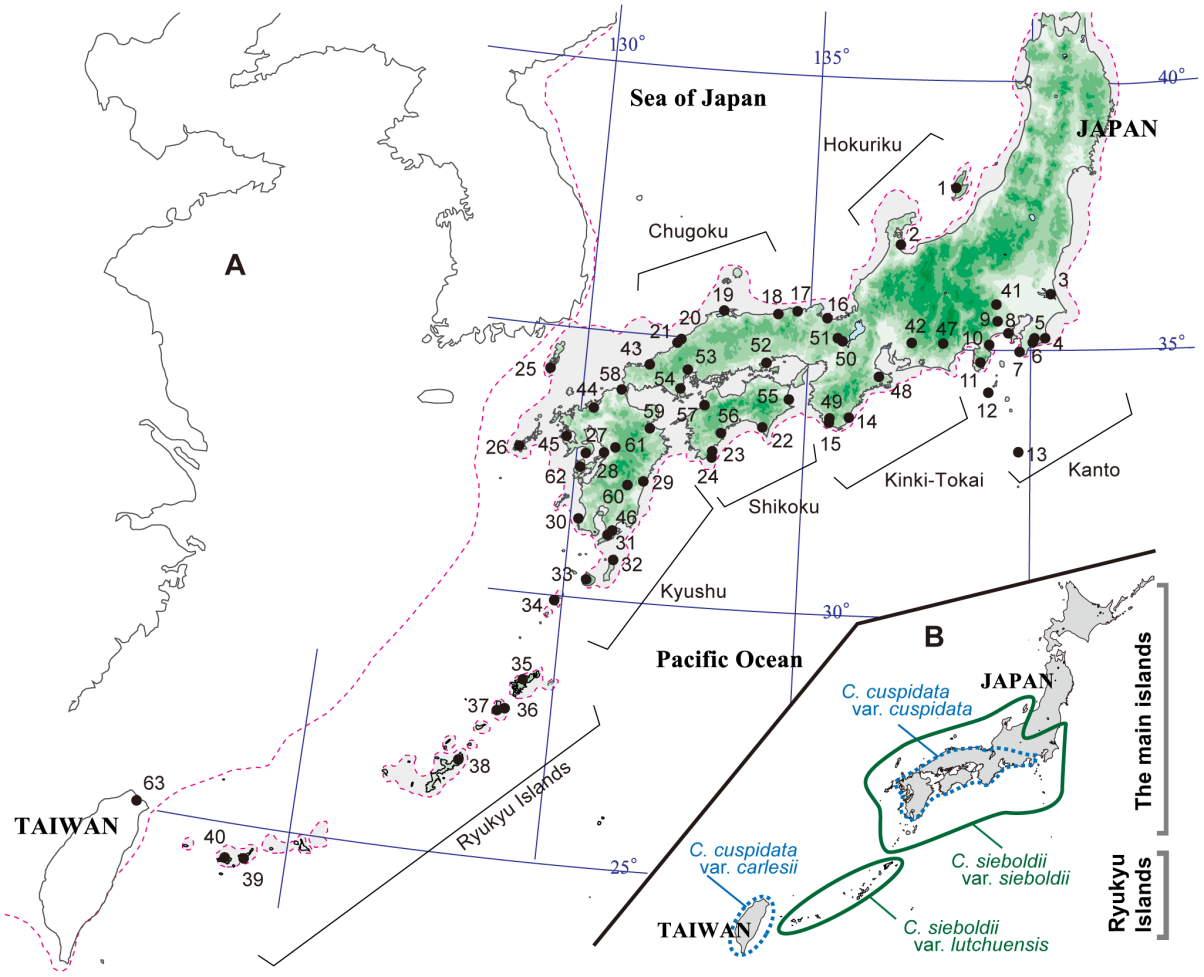
Locations of sampled populations. (A) Locations of the 63 *Castanopsis* populations sampled. Numbers correspond to the population numbers in Table S1. The dotted line indicates the coastline of the LGM about 18,000 to 24,000 years ago. (B) Distribution ranges of *Castanopsis* species and varieties in Japan and surrounding areas.

Palynological evidence has indicated that the broadleaved evergreen forests in Japan experienced cold periods at least four times during the Quaternary [2], [14]. During the glacial periods, climatic cooling caused the distribution of these forests to shift in a southerly direction and towards lower altitudes. The pollen record indicates that refugial populations of the broadleaved evergreen forests were restricted to southern areas, mainly at the southern end of Kyushu, and that they migrated northward from the refugia after the Last Glacial Maximum (LGM) [2], [15]. However, on the basis of historical climate data, some ecologists have proposed that these forests might have survived in multiple refugia along the Pacific coasts of the main islands (the main islands; see Fig. 1B) as well as at the southern end of Kyushu during the glacial periods [16]–[19]. Because of the relatively slow molecular evolution of chloroplast DNA [20]–[22] and the extremely low levels of intraspecific variation in the chloroplast DNA of Japanese broadleaved evergreen species [23], [24], there is very little phylogeographic data available on the plant species that currently inhabit these forests.

It is extremely important to reveal the genetic diversity and phylogeographical structure of the keystone species in the forests. We aimed to investigate *Castanopsis*, which is the dominant tree species in East-Asian broadleaved evergreen forests. In Japan, the plant genus *Castanopsis* holds two species, *C. sieboldii* and *C. cuspidata* [25], [26]. While these two species are sometimes distributed sympatrically in the main islands, most trees can be assigned to one of the two species based on phenotypes [25], [26]. However, intermediate morphologies exist especially at sites where the two species coexist. It has long been a debate topic among plant taxonomists and ecologists whether *C. sieboldii* var. *sieboldii* and *C. cuspidata* var. *cuspidata* are independent species and whether the intermediate morphologies are a natural hybrid. However, the extremely low levels of intraspecific variation in the chloroplast DNA, have made it difficult to elucidate their phylogenetic and phylogeographic relationships.

In the present study, we first aim to determine whether *C. sieboldii* and *C. cuspidata* can be clearly distinguished on the basis of genetic information as well as morphologically observed data. Second, we attempt to elucidate the genetic structure within each of the two species. The study presented in this work was performed to analyze Expressed Sequence Tag (EST)-associated microsatellite genetic variation in *Castanopsis*. Finally, these analyses of the dominant trees in the broadleaved evergreen forests of Japan are then combined with previous results on the phylogeographic patterns of the plants growing in the same climatic zone to reconstruct the history of the forests.

## Materials and Methods

### Ethics Statement

All necessary permits were obtained for the described sampling sites in verbal or written form. For sampling sites belonging to Japanese national forests, we obtained permits from regional forestry offices, and for the private sampling sites, permits was obtained from the owners. The University of Tokyo Chiba Forest and Kumamoto Prefectural Forestry Research Guidance Place also issued the permit. The plant materials did not involve endangered or protected species.

### Plant materials

In Japan, the plant genus *Castanopsis* (Fagaceae) is represented by two species, *C. sieboldii* and *C. cuspidata* [25], [26]. *Castanopsis sieboldii* is found in the main islands and the Ryukyu Islands in Japan, and is divided into two varieties, *sieboldii* and *lutchuensis* (Fig. 1B). *Castanopsis cuspidata* is found in the main islands of Japan, Taiwan and the mainland China, and is again divided into two varieties, *cuspidata* in Japan and *carlesii* in Taiwan and China. *Castanopsis sieboldii* var. *sieboldii* is mainly found in coastal regions, whereas *C. cuspidata* var. *cuspidata* is restricted to interior upland terrain [27]. While these two species are sometimes distributed sympatrically in the main islands, most trees can be assigned to one of the two species based on morphological differences in seed size, shape, and the structure of the leaf epidermis [25], [26]. *Castanopsis sieboldii* var. *sieboldii* has large, oblong seeds and a double layer of epidermal cells, while *C. cuspidata* var. *cuspidata* has small, globular seeds and a single layer of epidermal cells. Intermediate morphologies are often observed, especially at sites where the two species coexist [28].

We collected fresh or silica-gel-dried leaves from 63 *Castanopsis* populations (Table S1, Fig. 1A), including 56 populations of *C. sieboldii* var. *sieboldii* and *C. cuspidata* var. *cuspidata* from the main islands of Japan, six *C. sieboldii* var. *lutchuensis* populations from the Ryukyu Islands. We also collected one *C. cuspidata* var. *carlesii* population from Taiwan, because *C. cuspidata* consists of two varieties, *cuspidata* and *carlesii*. The locations in which we sampled populations covered most of the altitudinal and geographic natural distribution of *Castanopsis* in Japan (Fig. 1A).

*Castanopsis* is insect pollinated plant species, which is difficult to detect the fossilized pollen in the past. Therefore, only one fossilized pollen locality of *Castanopsis* trees at Ryukyu Islands existed at the LGM [29]. The fossilized pollen records of *Castanopsis*-type broadleaved evergreen tree genus (i.e., *Castanopsis*, *Lithocarpus*, *Myrica*, and, or *Podocarpus*) at the LGM existed in southwestern Kyushu (at the mean frequency of several to 10%) and northern Kyushu (several %) as well as at Ryukyu Islands (10%) [29].

### Leaf epidermis morphology

We examined the epidermis of the leaves from each sampled individual, since this is the most effective way of discriminating between *C. sieboldii* var. *sieboldii*, *C. cuspidata* var. *cuspidata*, and hybrids [25]. We examined the epidermal layers of leaves from 1,349 individuals collected from 56 populations in the main islands of Japan. Transverse sections of the leaves were prepared by cutting with a knife and examined under a light microscope as described by Kobayashi [30].

### EST-SSR analysis

Total DNA was extracted from each sample using a modified CTAB (hexadecyltrimethyl ammonium bromide) method [31], or according to the method of Doyle & Doyle [32] after removing polysaccharides from each leaf sample using HEPES buffer at pH 8.0 [33]. We determined the genotypes of each sample with respect to 32 pairs of nuclear microsatellite markers (expressed sequence tags-simple sequence repeats, EST-SSRs). Thirty-one of these pairs had been developed previously by Ueno and Tsumura [34], Ueno *et al*. [35] and Ueno *et al*. [36], [37], and the 32^nd^, QmC00288, was developed in the course of this work (forward primer tgaggtgccggaaaatgaagtaa; reverse primer cgacccatcaggattcgtacaag) (Table S2). The DNA at each EST-SSR locus was amplified with the QIAGEN Multiplex PCR Kit using the protocol provided by the manufacturer. PCR products were detected using a PRISM 3100 sequencer in conjunction with the GENESCAN software package, and genotype scoring was performed using the GENOTYPER software package (both supplied by Applied Biosystems).

### Genetic diversity and genetic differentiation

We determined the genotypes of 1,502 *Castanopsis* trees collected from 63 sites for the 32 EST-SSR loci. To evaluate the genetic diversity over all populations, we calculated the total number of alleles (*N*_A_), the average gene diversity within populations (*H*_S_) [38], and the observed heterozygosity (*H*_O_). We also calculated the fixation indices, *F*_IS_ [39], across all populations at each locus and over all loci to measure departures from Hardy-Weinberg equilibrium. The significance of the deviations of *F*_IS_ from zero and the linkage disequilibrium for all locus pairs was evaluated by permutation tests with sequential Bonferroni correction. We calculated coefficients of gene differentiation, *G*_ST_ [40], which is defined as *F*_ST_ in the case of multiple alleles, to determine relative genetic differentiation among populations.

We used the following parameters calculated from the allele frequencies at all loci analyzed to evaluate the genetic diversity within each population: the average number of alleles (*N*_A_), unbiased heterozygosity (*H*_E_) [38], and allelic richness (*R*_S_) [41] calculated using a minimum sample size of 17. We also calculated the frequencies of rare alleles (defined as alleles with a frequency <1% in the 63 populations that we collected), and the frequencies of private alleles (i.e. alleles that are unique to a single population of the 63 collected) in each population. These analyses were performed using MSA [42] and FSTAT version 2.9.3.2 [43]. To compare the geographical pattern of genetic diversity within the two *Castanopsis* species, we employed GIS program GRASS [44] and constructed the map of the genetic diversity. Elevation data were extracted from the WORLDCLIM dataset [45].

We measured the genetic diversity among *C. sieboldii* populations (Nos. 1–40), *C. cuspidata* populations (No. 47–63) and mixed populations (No. 41–46) from seven districts (see Fig. 1A) using five population genetic parameters: *N*_A_, *H*_E_, *R*_S_, rare allele frequency, and private allele frequency. We tested the significance of the effect of dividing to these districts on genetic differentiation using hierfstat [46].

To test for reductions in effective population size due to founding events or population bottlenecks, we used the heterozygosity excess method of Cornuet & Luikart [47]. We applied Wilcoxon's signed rank tests under the assumption of mutation-drift equilibrium in the infinite allele model (IAM) [48] and two-phase model (TPM, under which 70% of the mutations were assumed to occur under the stepwise mutation model) using BOTTLENECK version 1.2 [49]. Sequential Bonferroni correction was used to determine significance in the multiple tests.

We also analyzed the relationships between the genetic diversity within each population (*R*_S_) and the current environmental conditions in their habitats to examine the impact of local conditions on genetic diversity. This was done using a generalized linear model (GLM) created in R [50]. We used a Gaussian error distribution and an identity link function because response variable *R*_S_ has continuous values. The environmental factors included in the model were latitude, longitude, altitude, precipitation in the coldest three months, precipitation in the warmest three months, minimum temperature of the coldest month, and mean temperature of the warmest three months, as shown in Table S1 and extracted from the WORLDCLIM dataset [45]. The model with the lowest Akaike information criterion (AIC) value was selected as the final model.

To assess the proportion of variance in *F*_ST_ [39] attributable to genetic differences between *C. sieboldii* and *C. cuspidata*, and among groups of populations within *C. sieboldii* and *C. cuspidata*, hierarchical analyses of molecular variance (AMOVA) [51] were carried out for each locus using ARLEQUIN 3.5 [52]. The proportion of variance in each hierarchical class was tested by permuting individual genotypes.

### Detecting outlier loci

Because STRUCTURE analyses are not suitable for studying loci under selection, we carefully checked the neutrality of each locus. We compared the distribution of the *F*_ST_ values over all loci to their expected distributions under an island model with the assumption of neutrality using the LOSITAN program [53], based on fdist as described by [54]. To calculate approximate *P* values for each locus, 10,000 independent loci were generated and the simulated *F*_ST_ distribution was compared to the observed *F*_ST_ values. This made it possible to identify outliers in a one-step process by defining them as observed *F*_ST_ values falling outside the 99% confidence interval for the simulated group.

### Genetic structure

We estimated the genetic structure among populations by constructing a neighbor-joining (NJ) tree [55] based on *D*_A_ distances [56] between all pairs of populations using MSA and PHYLIP [57].

In addition, we used the Bayesian clustering method to elucidate the genetic structure among populations of *Castanopsis* and within *C. sieboldii* and *C. cuspidata*, and to infer the most appropriate number of subpopulations (*K*) using STRUCTURE version 2.2 [58]. Simulations were run 10 times for each value of *K* (1–10) with 300,000 Markov chain Monte Carlo sampling runs after a burn-in period of 500,000 iterations, using the admixture model under the assumption of correlated allele frequencies. The most appropriate cluster number (*K*) was selected using the criterion of Evanno *et al*. [59], which is based on Δ*K*. These analyses were performed using two data sets, one covering all 32 loci and the other containing only loci without outliers. We used multiple regression analyses to investigate the relationship between the membership values calculated for each individual using STRUCTURE and the number of epidermal cell layers in its leaves.

We assessed the presence of isolation-by-distance patterns in *C. sieboldii* and *C. cuspidata* by comparing genetic distances to geographic distances (*D*_A_) between pairs of populations. The significance of the associations between the two types of distance was determined by the Mantel test [60] with 10,000 permutations using SPAGeDi version 1.2 [61].

## Results

### Leaf epidermal morphology

**Figure 2:**
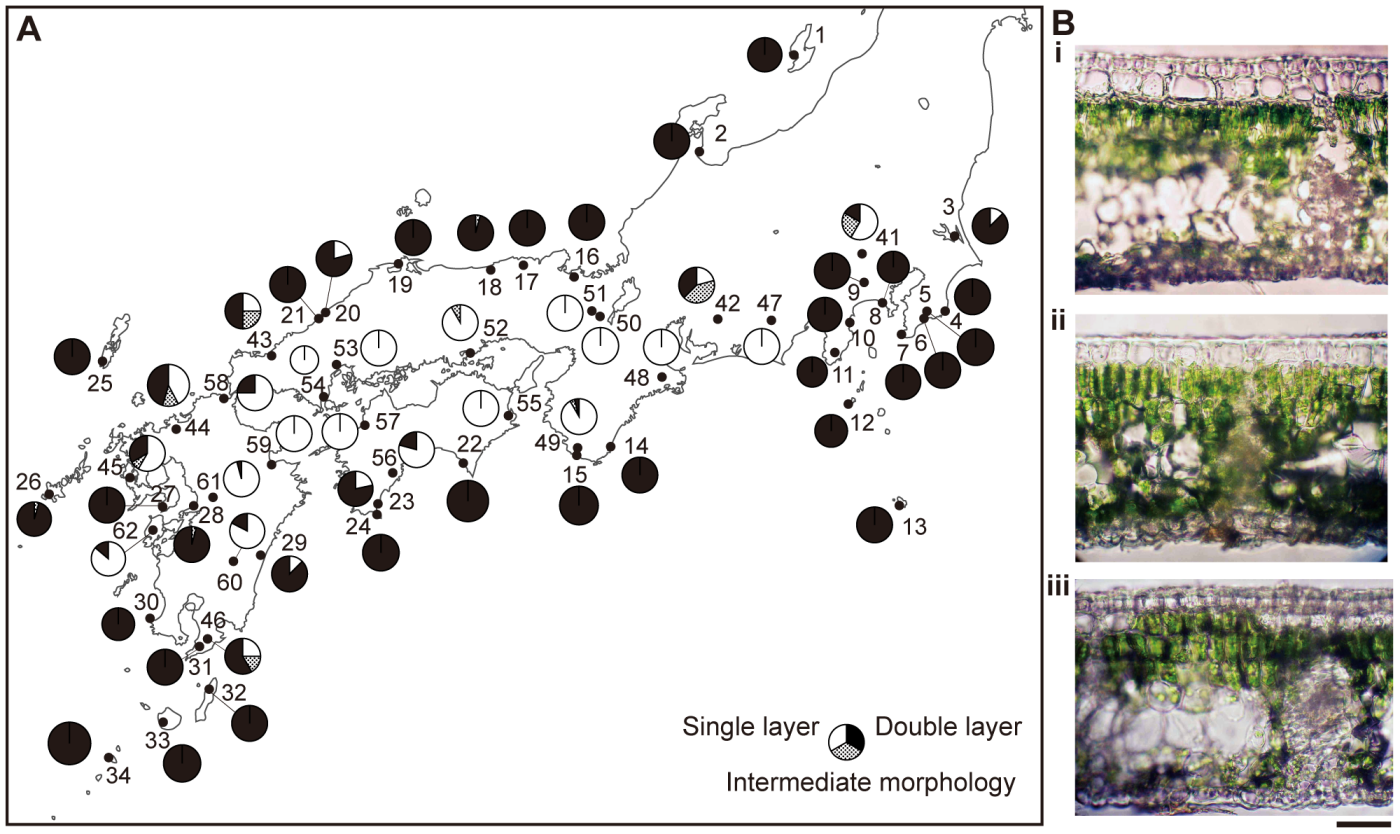
Leaf epidermis structure in *Castanopsis*. (A) Geographical distribution of leaf epidermis structure. Circle sizes are proportional to sample sizes. (B) Transverse sections of leaves; (i) a leaf taken from a tree growing in Maizuru (population no. 16) has a double epidermal cell layer, (ii) a leaf with a single epidermal cell layer from Ise (No. 48), (iii) intermediate epidermal morphology in a leaf from Hagi (No. 43). Scale bar = 20 µm.

Populations consisting mainly of individuals with single epidermal cell layers were distributed in inland areas, while populations in which individuals had double epidermal cell layers were distributed along the coastal areas (Fig. 2). These distributions are consistent with the geographic distribution of the two species, *C. cuspidata* var. *cuspidata* and *C. sieboldii* var. *sieboldii* (Fig. 1B). Individuals having both single and double epidermal cell layers within the same leaf (*i.e*. individuals with intermediate morphology) were primarily found in the six populations (Nos. 41–46) in which single- and double- epidermal cell layer individuals were sympatrically distributed, although one or two individuals with intermediate morphology were also found in five other populations (Nos. 18, 26, 28, 49, 52).

### Genetic diversity and genetic differentiation within *Castanopsis*

The EST-SSR loci were highly polymorphic: the total number of alleles detected over all populations at each locus ranged from 5 to 30, with an average value of 15.0 (Table S2). The average values of *H*_S_ and *H*_O_ over all loci were 0.644 and 0.591, respectively. Across all populations, the *F*_IS_ values deviated significantly and positively from zero at 12 loci, and over all loci. No evidence of significant linkage disequilibrium was detected in any of a total of 9,920 permutation tests for linkage disequilibrium between loci. High levels of genetic diversity within populations were also observed in each population (on average, *N*_A_ = 6.2, *H*_E_ = 0.644, *R*_S_ = 5.750, Rare allele = 0.275, Private allele  = 0.018; Table S1).

**Figure 3:**
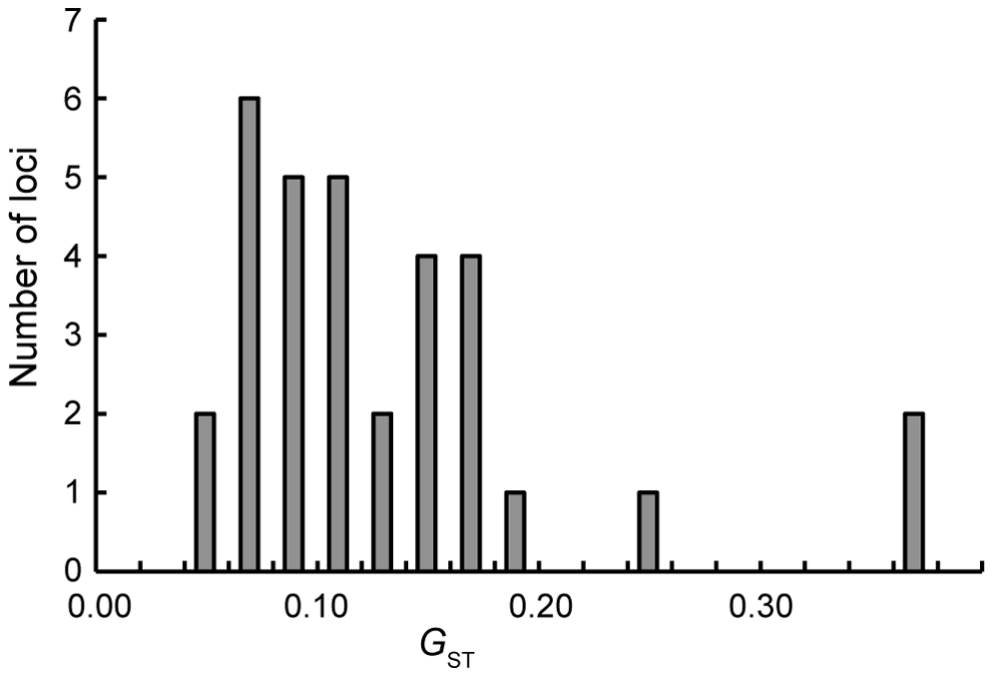
Distribution of *G*_ST_ values among 63 populations for 32 EST-SSR loci.

The overall genetic differentiation among populations at the 32 loci was low (*G*_ST_ = 0.122) for all *Castanopsis* populations. The *G*_ST_ value varied among loci, ranging from 0.053 at locus CcC02535 to 0.361 at locus CcC01513 (Fig. 3). AMOVA indicated that the proportion of variance among *C. sieboldii* and *C. cuspidata* populations and among populations within each species was 14.5% and 5.8%, respectively, and the *F*_ST_ value was 0.203 (*P*<0.001) (Table 1).

**Table 1:** AMOVA of genetic variation for *Castanopsis* and within *C. sieboldii* and *C. cuspidata* populations based on 32 EST-SSR markers.

Source of variation	df	Sum of squares	Variance components	% of total variation	Fixation indices	*P* value
Among species: [*C. sieboldii*, Nos. 1–40] [*C. cuspidata*, Nos. 47–63]	1	2105.0	1.85	14.5	*F*_CT_ = 0.145	*P*<0.001
Among populations within species	55	2479.1	0.74	5.8	*F*_SC_ = 0.068	*P*<0.001
Within populations	2643	26813.1	10.14	79.7	*F*_ST_ = 0.203	*P*<0.001
Total	2699	31397.1	12.73			
Among groups in *C. sieboldii*: [Ryukyu Islands] [main islands]	1	141.4	0.22	2.0	*F*_CT_ = 0.020	*P*<0.001
Among populations within groups	38	1624.3	0.69	6.5	*F*_SC_ = 0.067	*P*<0.001
Within populations	1876	18162.2	9.68	91.5	*F*_ST_ = 0.086	*P*<0.001
Total	1915	19927.9	10.59			
Among groups in *C. sieboldii*: [Ryukyu] [Kyushu, Chugoku, Shikoku] [Kinki-Tokai, Kanto, Hokuriku]	2	252.0	0.15	1.4	*F*_CT_ = 0.014	*P*<0.001
Among populations within groups	37	1513.8	0.65	6.2	*F*_SC_ = 0.063	*P*<0.001
Within populations	1876	18162.2	9.68	92.4	*F*_ST_ = 0.076	*P*<0.001
Total	1915	19927.9	10.48			
Among groups in *C. sieboldii*: [Ryukyu] [Kyushu, Chugoku] [Shikoku, Kinki-Tokai, Kanto, Hokuriku]	2	244.4	0.14	1.3	*F*_CT_ = 0.013	*P*<0.001
Among populations within groups	37	1521.3	0.66	6.3	*F*_SC_ = 0.063	*P*<0.001
Within populations	1876	18162.2	9.68	92.4	*F*_ST_ = 0.076	*P*<0.001
Total	1915	19927.9	10.48			
Among groups in *C. cuspidata*: [Taiwan] [Japan]	1	119.2	1.25	9.5	*F*_CT_ = 0.095	*P* = 0.053
Among populations within groups	15	594.1	0.61	4.6	*F*_SC_ = 0.051	*P*<0.001
Within populations	767	8650.9	11.28	85.9	*F*_ST_ = 0.141	*P*<0.001
Total	783	9364.3	13.13			
Among groups in *C. cuspidata*: [Kyushu] [Chugoku,Shikoku] [Kinki-Tokai]	2	71.2	−0.02	−0.2	*F*_CT_ = −0.001	*P* = 0.767
Among populations within groups	13	523.0	0.62	5.2	*F*_SC_ = 0.051	*P*<0.001
Within populations	732	8295.5	11.33	95.0	*F*_ST_ = 0.050	*P*<0.001
Total	747	8889.6	11.93			

### Outlier loci

**Figure 4:**
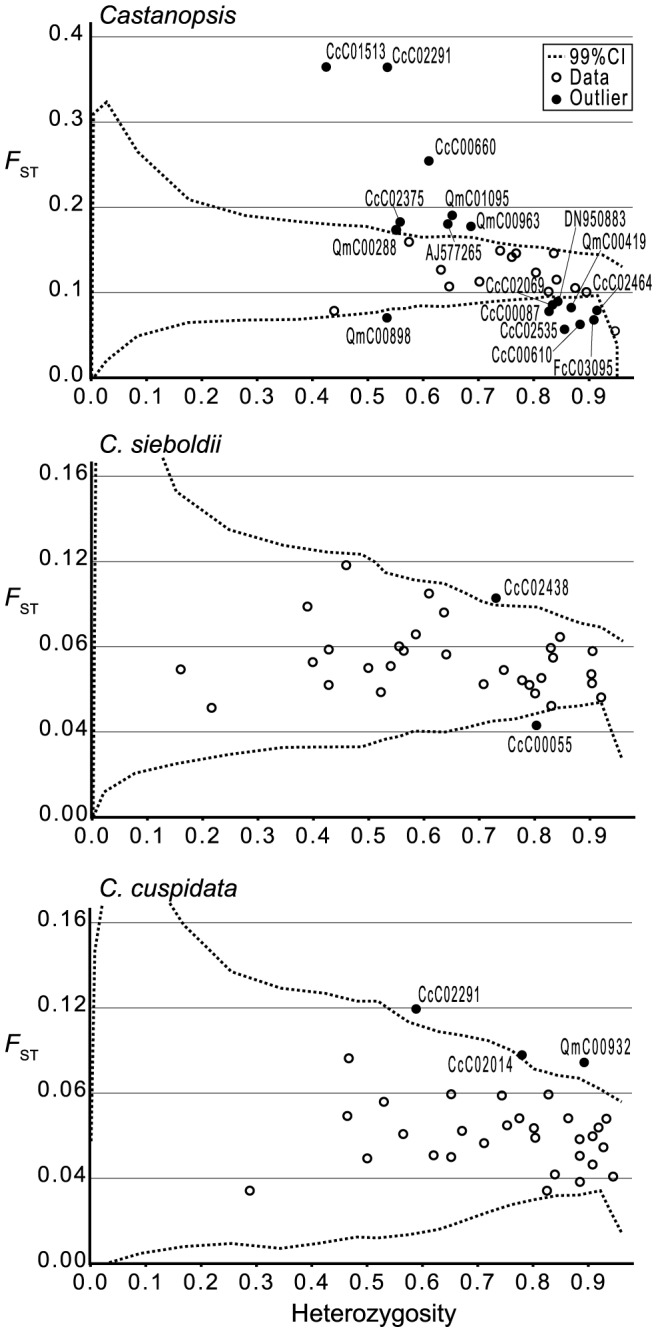
Distribution of *F*_ST_ values as a function of the within-population heterozygosity based on 32 EST-SSR loci.

In total, 21 loci were identified as outliers. Seventeen outlier loci were identified for all *Castanopsis* populations: eight of these had *F*_ST_ values exceeding the upper 99% CI (Fig. 4, Table S3) and the other nine had *F*_ST_ values below the lower 99% CI threshold. Two outlier loci were identified in the 937 individuals from the *C. sieboldii* populations with two epidermal cell layers. Three outlier loci were identified in the 368 individuals from the *C. cuspidata* populations with a single epidermal cell layer.

### Genetic structure within *Castanopsis*

**Figure 5:**
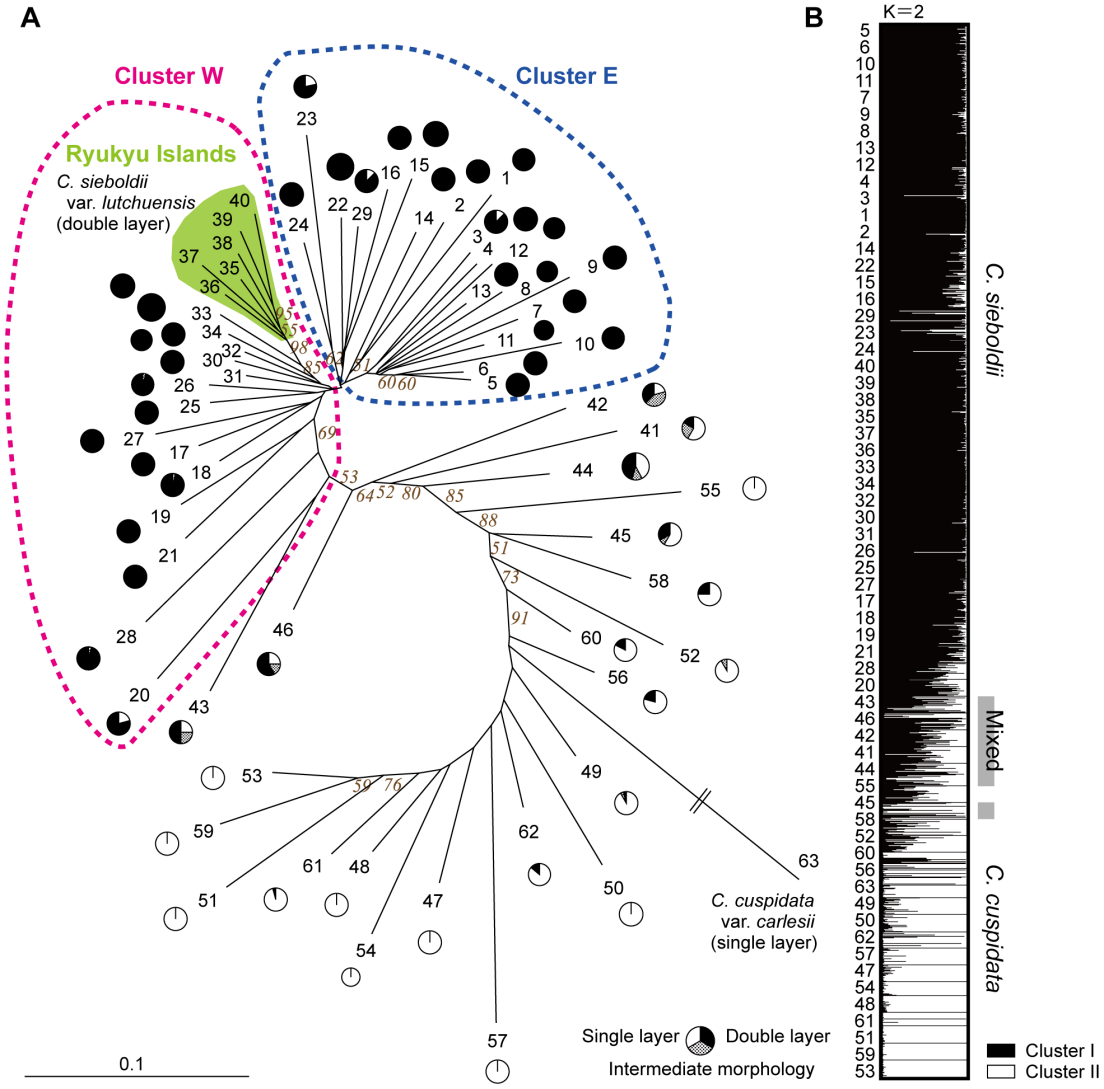
Genetic relationships among the 63 *Castanopsis* populations over 32 EST-SSR loci. In total, 1,502 individuals were surveyed in this study. Numbers correspond to the population numbers in Table S1. (A) Neighbor-joining tree based on Nei's genetic distances (*D*_A_) and the leaf epidermal type of each population (see Fig. 2). Values in italics are percentages of 1,000 bootstrap replicates supporting the respective nodes. (B) Distribution of cluster memberships at the individual and population levels estimated using STRUCTURE [58].

The NJ tree containing all identified *Castanopsis* populations revealed the presence of two distinct population groups, one consisting of individuals having a single epidermal cell layer (Nos. 47–63) and the other consisting of individuals having a double epidermal cell layer (Nos. 1–40) (Fig. 5A). Six populations containing individuals with both types of epidermis as well as intermediate epidermal morphologies (Nos. 41–46) were positioned between the two major clusters in the NJ tree.

Bayesian clustering of the information from the 32 loci demonstrated that the model with *K* = 2 provided a satisfactory explanation of the observed data (this simulation had the highest Δ*K* value). The results obtained when using data from only 11 loci and excluding 21 outliers (Fig. 4, Table S3) were almost identical to those obtained when considering the full set of 32. Membership in the two clusters correlated strongly with leaf morphology: individuals with a single epidermal cell layer generally belonged to one cluster and those with a double epidermal cell layer to the other. The populations containing individuals with intermediate epidermal morphology or with both morphologies were shown to represent admixtures of the two clusters mentioned above.

Multiple regression analysis showed that the Q values (which measure group membership) calculated for each individual using STRUCTURE correlated significantly with the number of cell layers in the epidermis of the leaves (*P*<0.001).

### Genetic diversity and genetic differentiation within *C. sieboldii* and *C. cuspidata*

**Figure 6:**
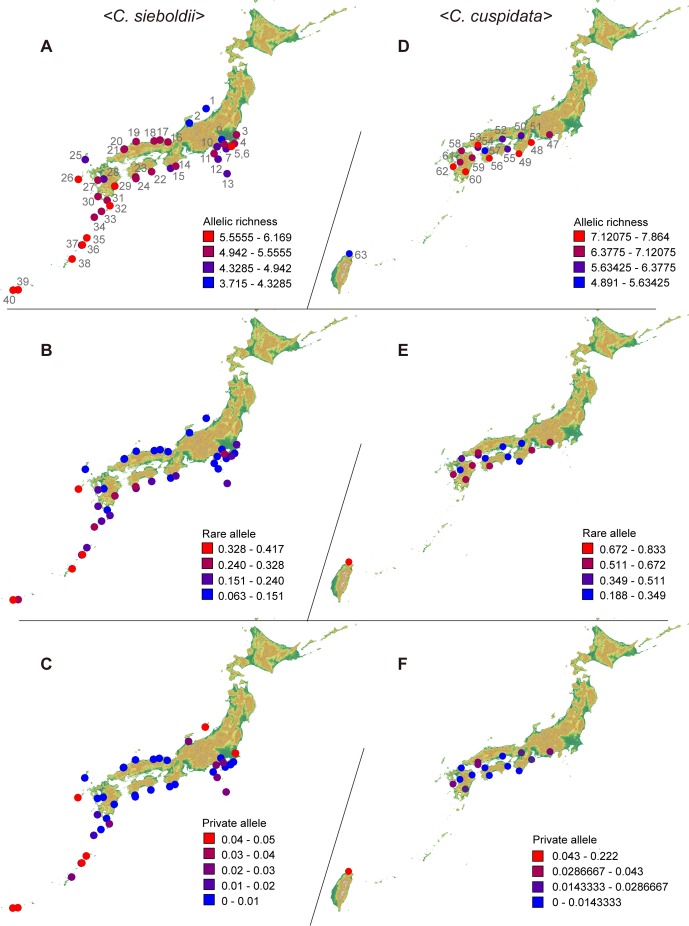
Maps of genetic diversity of *Castanopsis sieboldii* and *C. cuspidata* populations using GRASS [44]. (A) – (C) Allelic richness, the frequencies of rare alleles and private alleles of *C. sieboldii*. (D) – (F) Allelic richness, the frequencies of rare alleles and private alleles of *C. cuspidata*.

Populations of *C. cuspidata* had higher values of all five population genetic parameters than those of *C. sieboldii* (on average, *N*_A_ = 6.88 and 5.45, *H*_E_ = 0.69 and 0.59, *R*_S_ = 6.47 and 5.08, rare allele  = 0.53 and 0.17, private allele  = 0.07 and 0.02, Table 2). The significant effect on genetic differentiation was observed among regions in all populations (*P* = 0.01) and in *C. sieboldii* populations (*P* = 0.02), while no significant effect was observed in *C. cuspidata* populations (*P* = 0.35). Within *C. sieboldii* populations, those in western Japan (Kyushu) tended to have above average *N*_A_, *R*_S_, and rare allele values (Table 2 and Fig. 6). Populations of *C. sieboldii* var. *lutchuensis* from the Ryukyu Islands had the highest values for all of these parameters. Within *C. cuspidata* populations, populations in western Japan (Kyushu) tended to have above average *N*_A_ and *R*_S_ values (Table 2). The population of *C. cuspidata* var. *carlesii* in Taiwan was found to have the highest frequencies of rare alleles and private alleles (Fig. 6), whereas it had the lowest values of *N*_A_, *H*_E_, and *R*_S_. Mixed populations had higher values of *N*_A_ and *H*_E_.

**Table 2:** Genetic diversity of *Castanopsis sieboldii* and *C. cuspidata* populations observed in each district.

Species	Variety	District	Population No.	*N*_A_ a	*H*_E_^b^	*R*_S_^c^	Rare allele	Private allele
*Castanopsis sieboldii*	(var. *sieboldii*)	Hokuriku	1–2	4.11	0.50	3.91	0.07	0.03
		Kanto	3–13	5.34	0.60	5.04	0.16	0.01
		Kinki-Tokai	14–17	5.55	0.61	5.17	0.14	0.00
		Chugoku-Shikoku	18–24	5.70	0.61	5.25	0.14	0.00
		Kyushu	25–34	6.01	0.61	5.51	0.22	0.01
	(var. *lutchuensis*)	Ryukyu Is.	35–40	6.02	0.62	5.63	0.29	0.04
		Mean		5.45	0.59	5.08	0.17	0.02
*Castanopsis cuspidata*	(var. *cuspidata*)	Kinki-Tokai	47–51	7.22	0.70	6.66	0.39	0.02
		Chugoku-Shikoku	52–57	6.85	0.70	6.40	0.41	0.01
		Kyushu	58–62	7.78	0.72	7.24	0.51	0.01
	(var. *carlesii*)	Taiwan	63	5.66	0.63	5.59	0.83	0.22
		Mean		6.88	0.69	6.47	0.53	0.07
Mixed			41–46	7.10	0.72	6.53	0.36	0.03

a *N*_A_, Number of alleles; ^b^ *H*_E_, expected heterozygosity; ^c^ *R*_S_, Allelic richness.

According to Wilcoxon's signed rank tests, 15 populations of *C. sieboldii* (four from Kanto, two from Kinki-Tokai, four from Chugoku, one from Shikoku and two from the Kyushu and two from Ryukyu Islands), 15 populations of *C. cuspidata* (all but one of the *C. cuspidata* var. *cuspidata* populations), and all of the mixed populations deviated significantly from mutation-drift equilibrium under the IAM after sequential Bonferroni correction (Table S1). One population of *C. sieboldii* from Kanto, two of the Chugoku-Shikoku populations of *C. cuspidata* and one mixed population showed significant deviation under the TPM after sequential Bonferroni correction.

**Figure 7:**
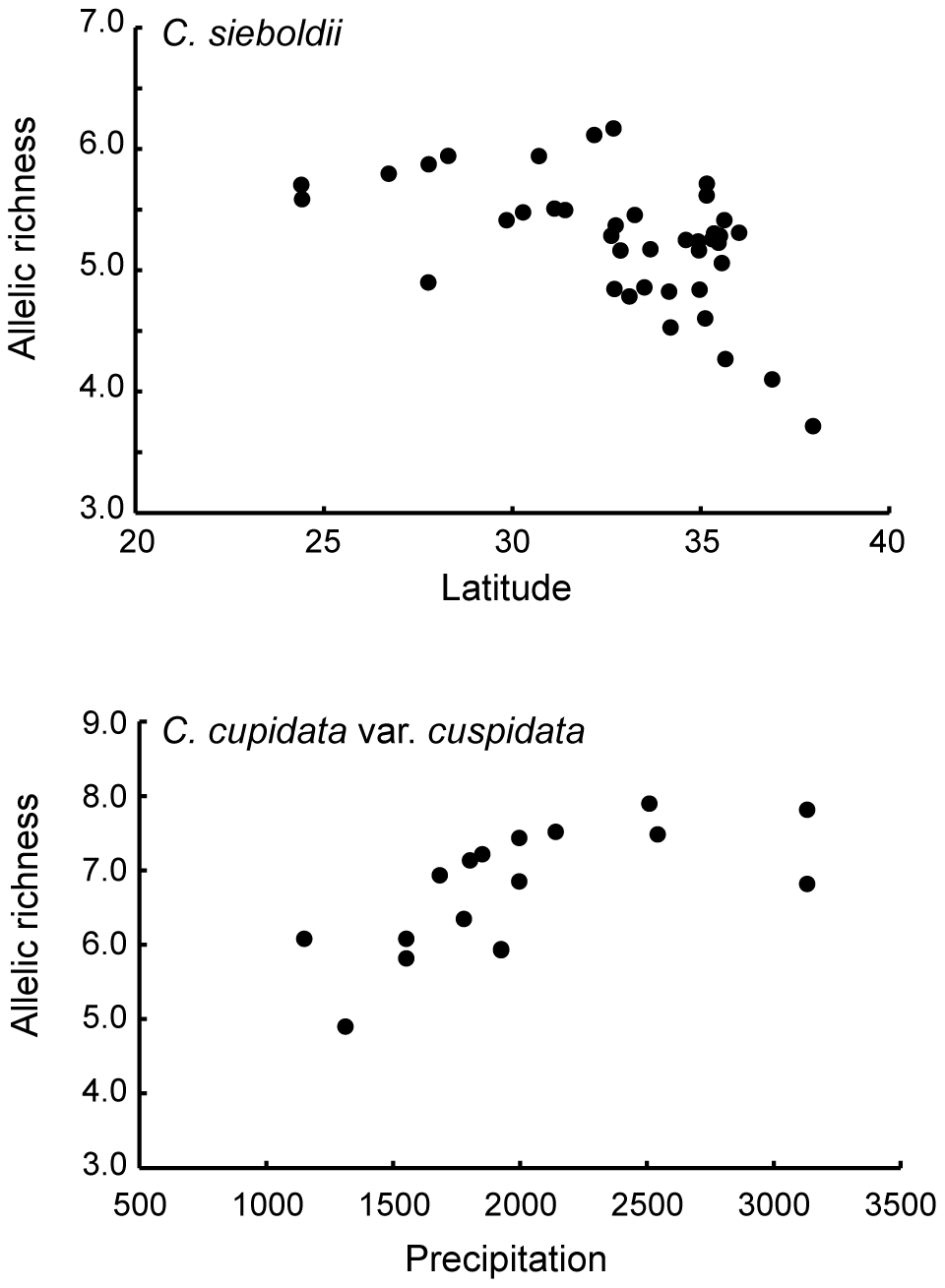
Relationships between the genetic diversity of *Castanopsis* and the current environmental conditions. Relationship between allelic richness and latitude for 40 populations of *C. sieboldii*, and between allelic richness and precipitation in the warmest three months for 16 populations of *C. cuspidata* var. *cuspidata*.

An analysis of variance for the selected GLM model using the AIC revealed that latitude significantly correlated with the genetic diversity (*R*_S_) of *C. sieboldii* populations (*R*_S_ = 11.066 – 0.168 Latitude, SE = 0.056, *t* = −2.964, *P*<0.01, Fig. 7), and *C. sieboldii* var. *sieboldii* populations (*R*_S_ = 10.891 – 0.168 Latitude, SE = 0.043, *t* = −3.879, *P*<0.001), and that precipitation in the warmest three months significantly correlated with the genetic diversity of *C. cuspidata* var. *cuspidata* populations (*R*_S_ = 15.52 – 0.082 Precipitation, SE = 0.0006, *t* = 3.299, *P*<0.01).

The overall genetic differentiation among populations at the 32 loci was low (*G*_ST_ = 0.069 and 0.057 among the *C. sieboldii* and *C. cuspidata* populations, respectively). AMOVA indicated that a small fraction of the observed gene diversity was attributable to differences between the various geographic regions in which *C. sieboldii* populations occur (1.3–2.0%) and differences among populations within groups (6.2–6.5%) (Table 1). In *C. cuspidata* populations, proportions of variance among groups and among populations within groups were 9.5 and 4.6%, respectively, while in *C. cuspidata* var. *cuspidata* populations, no significant level of gene diversity was attributable to differences between the geographic regions (−0.2%) and differences among populations within groups (5.2%).

### Genetic structure within *C. sieboldii* and *C. cuspidata*

**Figure 8:**
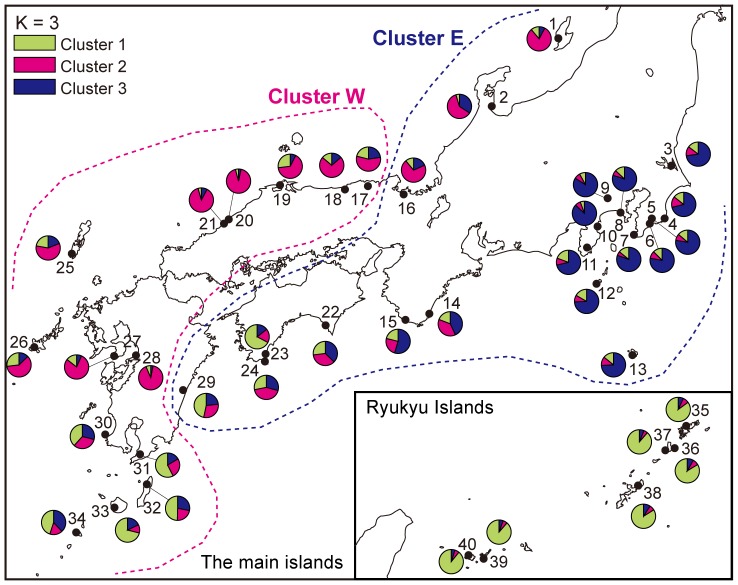
Genetic relationships among the 40 *Castanopsis sieboldii* populations estimated using STRUCTURE [58]. These data include 937 *C. sieboldii* individuals on 32 EST-SSR loci. Dotted lines indicate the distributions of clusters revealed by the NJ tree shown in Fig. 5.

The NJ tree of *C. sieboldii* contained two major clusters corresponding to populations from the western (Cluster W) and eastern (Cluster E) regions (Fig. 5A and dotted lines in Fig. 8). Within cluster W, populations from the Ryukyu Islands (*C. sieboldii* var. *lutchuensis*) clustered strongly together to form a sub-cluster.

A STRUCTURE analysis was performed, focusing exclusively on 937 individuals with a double-layered epidermis from 40 populations of *C. sieboldii*. Bayesian clustering demonstrated that the highest Δ*K* value was achieved when *K* = 3, therefore the fraction of ancestry derived from each of three clusters was estimated. We analyzed the STRUCTURE analysis both with and without the two outlier loci and the results were in general congruent between these analysis. The frequency of cluster 1 was particularly high among populations from the Ryukyu Islands (shown in green in Fig. 8) and it was also relatively high in the mainland populations from the south-western Pacific Ocean side of Japan. The frequency of cluster 2 (red) was higher in populations located on the Sea of Japan side than in the Pacific Ocean side. Cluster 3 (blue) was most common in the eastern parts (Kanto) but it was also present on the Pacific Ocean side.

In contrast, *C. cuspidata* demonstrated no clustering in the NJ tree (Figure 5A). Bayesian clustering of the information, focusing exclusively on 368 individuals with a single-layered epidermis from 17 population of *C. cuspidata*, demonstrated that the highest Δ*K* was when *K* = 3. We analyzed the STRUCTURE analysis both with and without the three outlier loci and the results were in general congruent between these analysis. One of the clusters was found in populations of Taiwan, and the other two clusters were found in populations of Japan. The geographic distribution of these two clusters in Japan showed no clear geographical structuring (data not shown).

**Figure 9:**
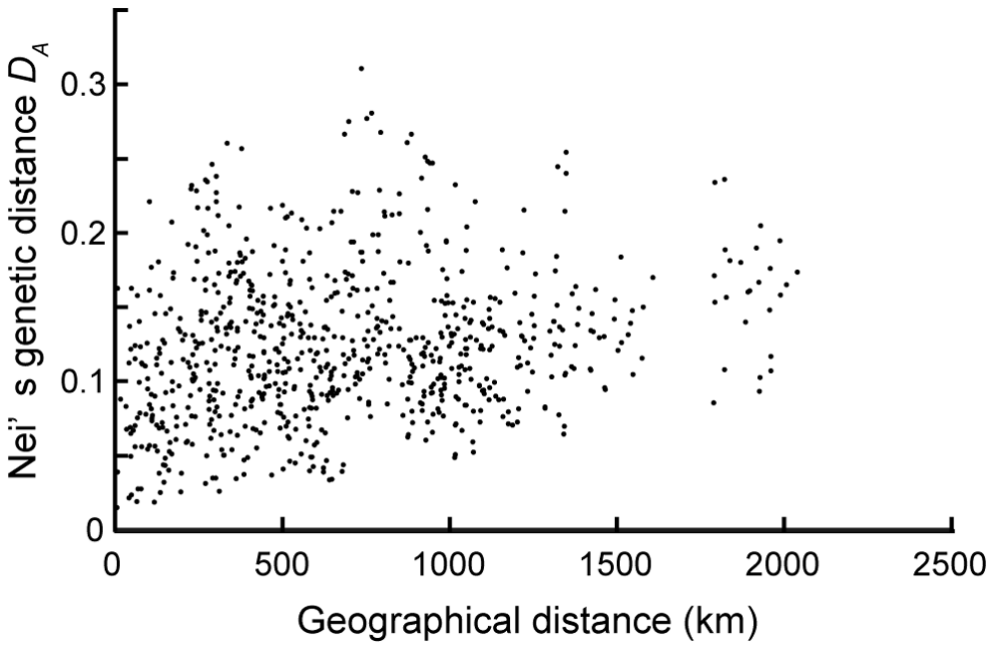
Relationship between geographical distance and Nei's genetic distance (*D*_A_) for 32 loci in *Castanopsis sieboldii*. The significance of this relationship, which represents the magnitude of isolation by distance, was tested using the Mantel test (*C. sieboldii*, *r* = 0.164, *P = *0.04; *C. sieboldii* var. *sieboldii*, *r* = 0.105, *P = *0.04).

A Mantel test revealed a weak but significant correlation between the pair-wise genetic distance and geographical distance between populations for *C. sieboldii* (*r* = 0.164, *P* = 0.04 within *C. sieboldii* and *r* = 0.105, *P* = 0.04 within *C. sieboldii* var. *sieboldii*. Fig. 9), while the correlation coefficients for *C. cuspidata* proved to be non-significant (*r* = 0.700, *P* = 0.05 within *C. cuspidata* and *r* = −0.211, *P* = 0.95 within *C. cuspidata* var. *cuspidata*, figure not shown).

## Discussion

### Genetic diversity in EST-SSR markers

We found low levels of genetic differentiation among the populations that we examined (*G*_ST_ = 0.069 in *C. sieboldii* populations, and *G*_ST_ = 0.057 in *C. cuspidata* populations). Wind-pollinated and wind-dispersed tree species have previously been reported to exhibit low values of *G*_ST_ (0.014–0.038 in *Fagus crenata*; 0.028–0.047 in *Cryptomeria japonica*), as reviewed by Tsumura [12]. In contrast, insect- or animal-pollinated and animal-dispersed tree species exhibit relatively high *G*_ST_ values: 0.144 in *Camellia japonica* [62] and 0.318 in *Zanthoxylum ailanthoides* [63]. The *G*_ST_ values of the *Castanopsis* species examined in this study were relatively low even though they are insect-pollinated and animal-dispersed. Yumoto [64] observed that *Castanopsis* attracts many taxonomic groups of insects, such as small bees, wasps, flies, beetles and butterflies. Moreover, flower-visiting insects have been observed to visit *Castanopsis* more often than other canopy-flowering tree species. This would be expected to result in relatively low levels of genetic differentiation among *Castanopsis* populations due to frequent large-scale gene flow.

The *F*_IS_ values across all populations at 12 loci and over all loci deviated significantly and positively from zero (Table S2). This may be due to population substructure, selection or the presence of null alleles, for which the last one is less likely to be a problem for EST microsatellites, but no specific hypothesis can be ruled out.

### Morphological and genetic differentiation between *C. sieboldii* and *C. cuspidata*

It has long been a debate topic whether *C. sieboldii* var. *sieboldii* and *C. cuspidata* var. *cuspidata* are independent species, although *Castanopsis* species are dominant important trees in the warm temperate zone of Japan. In this study, we solved the problem by adopting a collecting strategy designed to cover the whole distributional range of these two species, and analyzed a large quantity of both morphological data and EST-associated microsatellite data.

The Bayesian clustering analyses and the phylogenetic analysis revealed the presence of two well-separated groups of populations, one consisting of individuals with a single-layer epidermis and the other consisting of individuals with a dual-layer epidermis (Fig. 5). Moreover, at the individual level, the membership values obtained through STRUCTURE analyses correlated significantly with the number of epidermal layers in the leaves (*P*<0.001). The two clusters identified in our genetic clustering analyses were largely consistent in terms of their leaf epidermal structure, indicating that this morphological trait is a useful characteristic for discriminating between the two genetic groups of *Castanopsis*. These results suggest that the two clusters should be treated as independent species, i.e. *C. sieboldii* and *C. cuspidata*. A far larger proportion of the total variance (14.5%, *P*<0.001) (Table 1) was explained by differences between these two species than by variation within *C. sieboldii* (1.3 – 2.0%) and within *C. cuspidata* in Japan (−0.2%), further demonstrating their clear genetic differentiation.

Intermediate epidermal morphological type has been frequently reported, especially at sites where the two species are distributed sympatrically, and it has been considered to result from natural hybridization events [28], [65]. Individuals of intermediate epidermal morphology contained alleles from both *C. sieboldii* and *C. cuspidata*, and in consequence, the genetic diversity values of mixed populations (No. 41–46) were higher than those of the other populations (Table 2). The genetic structure observed in the six mixed populations in which single-, double-, and intermediate epidermal cell layer individuals were sympatrically distributed is an admixture of both species (Fig. 5). This suggested that the individuals with intermediate epidermal morphology were natural hybrids. In this study, *C. sieboldii* and *C. cuspidata*, and their hybrids, were found to be distinguishable using both the morphological character and genetic information, even though natural hybrids are formed. These results suggest that hybrids may have reduced fitness maybe due to outbreeding depression [66].

Our results show that, where the two species do not coexist, there is clear differentiation between *C. sieboldii* var. *sieboldii* and *C. cuspidata* var. *cuspidata*. The habitats of these two species are to some degree differentiated: *C. cuspidata* forests predominate in low altitude mountains in inland areas, while *C. sieboldii* var. *sieboldii* forests are distributed in the Ryukyu Islands as well as coastal regions in the main islands, which are often exposed to a strong onshore wind. The morphological characters of these two species, for example leaf epidermal structure and seed size and shape, may have diverged in order to adapt to the different environments in these habitats. In future work, we will analyze the phylogeny and morphology of *Castanopsis* distributed throughout East Asia, and discuss the diversification of *Castanopsis* in Japan.

### Genetic structure and diversity within *C. sieboldii*

The Bayesian and NJ tree clustering analysis of *C. sieboldii* defined three groups and provided clear indications of genetic divergence among the populations from the Ryukyu Islands and the western and eastern parts of the main islands (Figs. 5, 8). The AMOVA showed that these three groups are genetically differentiated (1.3 – 2.0%, *P*<0.001) (Table 1). These results indicate that there are three major lineages of the nuclear genome in *C. sieboldii -* one distributed on the Ryukyu Islands that corresponds to *C. sieboldii* var. *lutchuensis*, and two, distributed in the western and eastern parts of the main islands, that correspond to *C. sieboldii* var. *sieboldii*. In microsatellite analyses of *Castanopsis* using seedlings grown from seeds by Yamada et al (2006), clusters within *C. sieboldii* were not clearly differentiated. This was probably due to the extremely small number of mother trees (5–7) sampled from each population and to the limited geographical dataset (no samples were taken from along the Pacific coast). Our collecting strategy covered the distributional range of the species, and most populations, in areas where *C. cuspidata* does not coexist, exhibited clear genetic differentiation of three groups in *C. sieboldii*.

The greatest levels of genetic diversity in *C. sieboldii* are observed in the populations of the Ryukyu Islands (Table 2 and Fig. 6), suggesting that these populations have remained sufficiently large for ancestral polymorphism to be retained from the glacial periods up to the present day. In the Ryukyu Islands, genetic uniqueness and high genetic diversity have been found in several other plant [11], [67] and animal [68], [69] species that cohabit in warm-temperate and subtropical zones of Japan. The fossilized pollen records demonstrate the existence of broadleaved evergreen trees in the Ryukyu Islands at the LGM [29]. It is likely that *C. sieboldii* var. *lutchuensis* survived the LGM without reduction in population size on these islands, because they are located far to the south of the main islands and thus have a much warmer climate.

The allelic richness within populations significantly decreased in more northerly populations (Fig. 7). The allelic richness of *C. sieboldii* var. *lutchuensis* from the Ryukyu Islands made a major contribution to this result, but a significant decrease was still detected within *C. sieboldii* var. *sieboldii* from the main islands. Moreover, higher genetic diversity was observed in the Kyushu region (Table 2 and Fig. 6). These results are consistent with the available fossil pollen data for *Castanopsis*-type forests during the LGM in the southern areas [15] and suggest the existence of refugia for *Castanopsis* forests in southern Kyushu at that time.

Both the NJ tree and the Bayesian clustering analysis of *C. sieboldii* indicated that there was genetic differentiation between the western and eastern populations in the main islands. A similar west-east genetic differentiation is also found in several plant and animal species inhabiting warm temperate zones in Japan (reviewed in [13]). The west-east genetic differentiation observed in *C. sieboldii* and other component species of the broadleaved evergreen forests implies that they have been isolated from each other in the western and eastern populations for an extended time, at least as far back as the LGM, and suggests the existence of eastern refugia. In addition to the eastern refugia, the relatively high number of private alleles in the Hokuriku region (Fig. 6), the northern limit of the distribution area along the Sea of Japan, is consistent with fossil pollen evidence of *Cryptomeria* survival in this location [70]. The result suggests that *Castanopsis* forests could have been survived also in northern small refugia along the Sea of Japan coasts.

### Genetic structure and genetic diversity within *C. cuspidata*

Bayesian clustering analysis indicated that there was genetic differentiation between the *C. cuspidata* var. *carlesii* population located in Taiwan and *C. cuspidata* var. *cuspidata* populations distributed in Japan. The fact that the highest values of rare allele and private allele richness were also observed in Taiwan (Table 2 and Fig. 6) provided further evidence for the genetic uniqueness of the *C. cuspidata* var. *carlesii* population.

Within *C. cuspidata* var. *cuspidata* populations distributed in Japan, no clear geographical genetic differentiation was found by the NJ tree, Bayesian and the AMOVA analyses (Fig. 5 and Table 1). The results may be due to the limited geographical distribution of *C. cuspidata* var. *cuspidata*.

Genetic diversity in the populations of *C. cuspidata* was on average higher than the values for *C. sieboldii* (Table 2, Fig. 7) but recent bottleneck effects were clearly detected for almost all populations (Table S1). *Castanopsis cuspidata* var. *cuspidata* forests predominate in low altitude inland areas, and natural populations of the species have therefore been fragmented due to recent human activities and are now present only in small areas around shrines and temples (Table S1). Population fragmentation may prevent gene flow among populations, resulting in a lack of significant correlation between genetic distance and geographic distance. The significant bottlenecks observed in almost all populations of *C. cuspidata* var. *cuspidata* may have been caused by isolation from other populations and recent reductions in population size, although these populations still have high levels of genetic diversity.

The allelic richness within populations of *C. cuspidata* var. *cuspidata* was significantly greater in those populations that experience a larger amount of precipitation in the warmest three months (Fig. 7). *Castanopsis cuspidata* var. *cuspidata* is distributed around the Seto Inland Sea, an area with a drier climate; however, the populations around the Seto Inland Sea are fragmented and small. The overall significant correlation between genetic diversity and rainfall was strongly impacted by the low diversity of the Seto inland sea populations. Larger values of allelic richness and many rare alleles were observed in the southern region, Kyushu (Table 2), a finding which is again consistent with the fossilized pollen data for *Castanopsis* in southern Kyushu during the LGM [15].

## Conclusions

Our study attempted to resolve a debate topic about systematics of a keystone species of Japanese forests and concluded that *C. sieboldii* var. *sieboldii* and *C. cuspidata* var. *cuspidata* are independent species. Our collecting strategy designed to cover the whole distributional range of these two species, and analyses of a large quantity of both morphological data and EST-associated microsatellite data enabled us to specify clear genetic differentiation between them. The west-east genetic differentiation observed in *C. sieboldii* on the main islands, a pattern which is also found in several plant and animal species inhabiting *Castanopsis* forests in Japan, suggests the existence of eastern refugia. This study has implication for conservation of an extremely important keystone species of Japan's forests.

## Supporting Information

Table S1 **Details of the** ***Castanopsis*** **populations investigated, and the population genetic parameters based on 32 EST-SSR markers.**
(DOC)

Table S2 **Polymorphisms for each of the 32 EST-SSR loci investigated in this study based on 63** ***Castanopsis*** **populations.**
(DOC)

Table S3 **Outlier loci (** ***P*** **<0.01) among** ***Castanopsis*** **populations and within** ***C. sieboldii*** **and** ***C. cuspidata*** **populations.**
(DOC)
