# Supplementary material for: Genetic Differentiation and Genetic Diversity of Castanopsis (Fagaceae), the Dominant Tree Species in Japanese Broadleaved Evergreen Forests, Revealed by Analysis of EST-Associated Microsatellites
Source: PLoS One. 2014 Jan 30;9(1):e87429. doi: 10.1371/journal.pone.0087429 (PMC3907500; doi:10.1371/journal.pone.0087429)
Supplement: Table S1 — Details of the Castanopsis populations investigated, and the population genetic parameters based on 32 EST-SSR markers. (DOC) [file pone.0087429.s001.doc]

**Table S1.** Details of the *Castanopsis* populations investigated, and the population genetic parameters based on 32 EST-SSR markers.

^a^ *N*_A_, Number of alleles; ^b^ *H*_E_, expected heterozygosity; ^c^ *R*_S_, Allelic richness

^d^ Probabilities associated with Wilcoxon’s signed rank tests after sequential Bonferroni correction to determine significance in the multiple tests are shown under an infinite allele model (IAM) and a two-phase model (TPM) for population bottleneck. **P* < 0.05; ***P* < 0.01; ****P* < 0.001

^e^ Leaf epidermal types are indicated as follows: C, all individuals have a single layer of epidermal cells; C-type, almost all individuals have a single epidermal cell layer; Mix, individuals with intermediate epidermal morphology and with both morphologies present within the population; S-type, almost all individuals have a double layer of epidermal cells; S, all individuals have a double layer of epidermal cells (see Fig. 2)

^f^ Forest types are indicated as follows: Pr, close to primeval forest, i.e., comparatively large forest in which diverse species still remained; Nt, Natural forest, i.e., small patches of shrine or temple forest in which no periodic clear cutting has occurred but where there has been occasional , low-impact human disturbance as well as a high degree of fragmentation by human development; Sc, secondary forest, i.e., coppices that were used as a source of firewood and charcoal
