# Supplementary material for: Genetic Differentiation and Genetic Diversity of Castanopsis (Fagaceae), the Dominant Tree Species in Japanese Broadleaved Evergreen Forests, Revealed by Analysis of EST-Associated Microsatellites
Source: PLoS One. 2014 Jan 30;9(1):e87429. doi: 10.1371/journal.pone.0087429 (PMC3907500; doi:10.1371/journal.pone.0087429)
Supplement: Table S2 — Polymorphisms for each of the 32 EST-SSR loci investigated in this study based on 63 Castanopsis populations. (DOC) [file pone.0087429.s002.doc]

**Table S2.** Polymorphisms for each of the 32 EST-SSR loci investigated in this study based on 63 *Castanopsis* populations.

^a^ Multiplex PCR primer combinations were as follows: locus No. 2, 11, 13, 16, 18 and 21 as set 1, locus 10, 15, 19, 24, 25, 31 and 32 as set 2, locus 3, 5, 6, 7 and 22 as set 3, locus 1, 9, 12, 14, 26, 27 and 30 as set 4, and locus 4, 8, 17, 20, 23, 28 and 29 as set 5

^b^ *N*_A_, Number of alleles; ^c^ *H*_O_, observed heterozygosity; ^d^ *H*_S_, average gene diversity within populations

^e^ The significance of *F*_IS_ values after sequential Bonferroni correction is indicated by asterisks (**P* < 0.05)
