# Supplementary material for: Genetic Differentiation and Genetic Diversity of Castanopsis (Fagaceae), the Dominant Tree Species in Japanese Broadleaved Evergreen Forests, Revealed by Analysis of EST-Associated Microsatellites
Source: PLoS One. 2014 Jan 30;9(1):e87429. doi: 10.1371/journal.pone.0087429 (PMC3907500; doi:10.1371/journal.pone.0087429)
Supplement: Table S3 — Outlier loci ( P <0.01) among Castanopsis populations and within C. sieboldii and C. cuspidata populations. (DOC) [file pone.0087429.s003.doc]

**Table S3.** Outlier loci (*P* < 0.01) among *Castanopsis* populations and within *C. sieboldii* and *C. cuspidata* populations.
